# Molecular Mechanisms in the Pathogenesis of Alzheimer’s disease and Tauopathies-Prion-Like Seeded Aggregation and Phosphorylation

**DOI:** 10.3390/biom6020024

**Published:** 2016-04-28

**Authors:** Masato Hasegawa

**Affiliations:** Department of Dementia and Higher Brain Function, Tokyo Metropolitan Institute of Medical Science; Setagaya-ku 156-8506, Japan; hasegawa-ms@igakuken.or.jp; Tel.: +81-3-6834-2349

**Keywords:** prion 2, amyloid 3, fibril 4, APP 5, *MAPT*, synuclein, TDP-43

## Abstract

Neurofibrillary tau pathology (tangles and threads) and extracellular amyloid-β (Aβ) pathology are defining features of Alzheimer’s disease. For 25 years, most research has focused on the amyloid hypothesis of AD pathogenesis and progression. But, because of failures in clinical trials of Aβ-targeted therapies and the new concept of prion-like propagation of intracellular abnormal proteins, tau has come back into the spotlight as a candidate therapeutic target in AD. Tau pathologies are found in a range of neurodegenerative disorders, but extensive analyses of pathological tau in diseased brains has demonstrated that the abnormal tau protein in each disease is structurally distinct, supporting the idea that progression of the diverse but characteristic tau pathologies occurs through prion-like seed-dependent aggregation. Therefore, intervention in the conversion of normal tau to abnormal forms and in cell-to-cell transmission of tau may be the key to development of disease-modifying therapies for AD and other dementing disorders.

## 1. Introduction

Alzheimer’s disease (AD) is neuropathologically characterized by the presence and spreading of two abnormal protein structures: so-called neurofibrillary tau pathologies (tangles and threads) and amyloid-β (Aβ) plaques in the neocortex. The distribution of tau pathology is closely correlated with disease symptoms and progression that can be used for classification of the six disease stages, while the Aβ pathology varies widely [1]. In 1991, pathogenic mutations in the amyloid protein precursor (APP) in rare cases of familial AD were discovered [2], and the amyloid cascade hypothesis (amyloid hypothesis) was proposed [3,4,5]. According to this hypothesis, production of toxic Aβ aggregates is the major pathway in the pathogenesis of AD. The discovery of AD-related mutations in presenilin 1 and 2 [6,7,8], which are catalytic subunits of the γ-secretase complex involved in APP cleavage and Aβ production [9,10,11], further supported the hypothesis. Nevertheless, there has been much debate concerning which of the pathologies is characteristic of AD, whether tau or Aβ is more important for the pathogenesis, what the relation is between tau and Aβ, what are the most toxic species of these proteins, and so on. The amyloid hypothesis is still widely accepted, but recent clinical and experimental results have raised serious questions [12,13,14]. In particular, the failure of a clinical study of Aβ vaccination, immunotherapies with the antibodies, and other studies with Aβ-targeted drugs strongly suggest that the amyloid hypothesis at least does not provide a sufficient basis for development of therapeutic drugs [15,16,17,18,19]. Interestingly, the clinical trial of Aβ vaccination showed that elimination of Aβ had no effect on the spread of tau pathologies or the progression of AD [15]. These results suggest that we need to develop a different therapeutic strategy for AD. Indeed, some tau-targeted therapies are already under clinical trial. Moreover, evidence for the prion-like mechanism has now spread to the major intracellular abnormal proteins including tau, as nicely reviewed in several articles [20,21,22,23,24]. In this review, I will focus on the formation and spread of abnormal tau, and the relationship between tau and Aβ in AD. I will also discuss the phosphorylation and the differences among abnormal tau forms found in AD and in other related dementias.

## 2. Abnormal Tau in AD Brain

Tau is one of the microtubule-associated proteins that promote assembly of tubulin to microtubules and stabilize them. As a cytoskeletal protein, it is expressed mainly in neurons, which develop long processes such as axons and dendrites for neuronal transmission. The human tau gene is localized in the region of 17q21 on the long arm of chromosome 17. Six tau isoforms are expressed in the adult human brain as a result of mRNA alternative splicing in various combinations, with or without exons 2, 3, and 10 [25]. Insertion of exon 10 affords 4-repeat (4R) tau isoforms, while 3-repeat (3R) tau isoforms are produced without exon 10. In adult human brain, almost equimolar amounts of 3R and 4R tau isoforms are expressed. Tau is localized mainly in axons, but is also present in the somatodendritic compartment in a phosphorylated state. Tau is a natively unfolded protein; it is highly soluble in water and stable on boiling treatment. This heat stability can be utilized to purify tau relatively easily from E. coli, cultured mammalian cells, and brain tissues [26,27].

**Figure 1:**
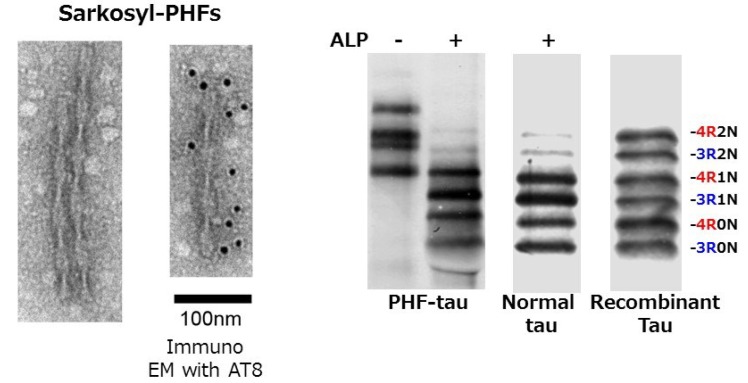
EM pictures of paired helical filaments in Sarkosyl-insoluble fraction prepared from AD brain, and the immunoblot analysis of tau before and after alkaline phosphatase (ALP) treatments, compared with soluble human tau and six recombinant human tau isoforms.

In AD brains, tau is accumulated in a hyperphosphorylated state in the pathological inclusions [28,29,30]. Ultrastructurally, it is observed as a unique filamentous structure, paired helical filaments (PHFs), which are fibrils/filaments of 10 nm diameter with 80 nm periodicity (Figure 1) [31,32,33,34,35,36,37,38].

**Figure 2:**
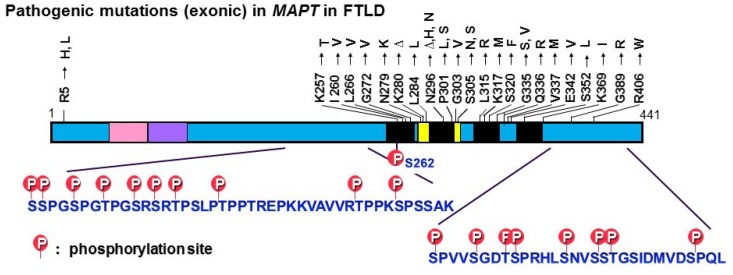
Exonic mutations in the tau gene (*MAPT*) reported in FTDP-17T and major tau phosphorylation sites identified in PHF-tau from AD brains. Most of the pathogenic mutations are located in the microtubule binding regions, whereas the phosphorylation sites are located outside these regions.

These structures are referred to as neurofibrillary tangles (NFTs) if they are formed in neuronal cell bodies, while they are referred to as threads if they are formed in dendrites or axons. Both are basically bundles of PHFs or related straight filaments (SFs). So, what is the difference between normal tau and abnormal tau in PHFs? In the early 1990s, we purified normal soluble tau and abnormal insoluble tau from AD brains, and extensively investigated whether there is any difference between them by means of peptide mapping, protein sequencing, and mass spectrometry. We found that abnormal tau in PHFs is highly phosphorylated at ~20 serine/threonine residues outside the microtubule binding region (Figure 2), partially deamidated at asparagine residues and partially ubiquitinated at lysine residues in the repeat regions [39,40,41,42,43].

Almost equal amounts of 3R and 4R tau isoforms were detected in the insoluble fraction of AD brains. Goedert *et al*. reported that pathological insoluble PHF-tau proteins could be separated into six tau isoforms after complete dephosphorylation [44]. These results suggested that abnormal phosphorylation might be the cause of the assembly and deposition of PHF [45,46,47,48].

## 3. Tau-Related Neurodegeneration and Dementias

Tau pathologies are also seen in other neurodegenerative dementing disorders, such as Pick’s disease (PiD), progressive supranuclear palsy (PSP), corticobasal degeneration (CBD), argyrophilic grain disease (AGD), tangle-only dementia, and chronic traumatic encephalopathies [49,50,51,52,53,54]. Because the Aβ pathology is relatively specific for AD and aging, it has been considered that tau pathology may not be the cause of dementia, but rather may be a non-specific consequence or a final common pathway of degenerating neurons. Indeed, some researchers ironically remarked that it is nonsense to investigate gravestones. However, in 1998, mutations in the tau gene (*MAPT*) were discovered in a familial form of dementia called frontotemporal dementia and parkinsonism linked to chromosome 17 (FTDP-17) [55,56,57]. Since then, many exonic and intronic mutations have been reported in familial and sporadic cases of dementia with tau accumulation [20,28,58]. These findings clearly demonstrated that tau abnormalities cause accumulation of tau and degeneration of neurons [59,60], and led to an increased focus on tau in research on AD and other dementing disorders. It should be noted that most of the missense tau mutations are located in the microtubule binding regions, whereas the phosphorylation sites are located outside these regions (Figure 2).

Although tau pathology can be seen in many dementing disorders, it is characteristic for each disease and can be used for classification of the disease phenotypes. Thus, there are many dementias that do not show Aβ pathology, and it is reasonable to think that abnormal tau may be the cause of these disorders. Therefore, we investigated abnormal tau proteins in these disorders, as well as in AD, in order to clarify what is the most important and common feature of the abnormal tau, and what the differences are among these diseases. We found that tau is accumulated as amyloid-like filamentous or fibrous structures in all these diseases, although the structures are different among the diseases [61]. The common features and differences in pathological tau among the diseases are discussed below.

## 4. Relationship between Tau Aggregation and Phosphorylation

What is the cause of tau aggregation or tau fibril formation? It is widely considered that hyperphosphorylation of tau triggers self-assembly, but is this true? Tau is accumulated in a hyperphosphorylated state in brains with AD and other degenerative diseases, and anti-phosphorylated tau antibodies are widely used for detecting abnormal tau pathologies, even in the early stages. Phosphorylation of tau reduces its interaction with tubulin, which may promote self-assembly. However, when we attempted to make PHFs from purified tau by phosphorylation of recombinant tau or tau purified from postmortem brains with various kinases, we failed to recapitulate self-assembly of tau into amyloid-like fibrils. Tau is a phosphoprotein and ~10 major phosphorylation sites have been identified in tau isolated from fetal and adult brains, although the stoichiometry is variable among the sites and is also dependent on the developmental stage [62]. Interestingly, tau in PHFs remains hyperphosphorylated for several to 10s of hours postmortem, whereas most phosphorylated proteins are dephosphorylated within 30 minutes after death [63]. So, why is tau in PHF not rapidly dephosphorylated? The answer is that it is aggregated in the form of stable filamentous structures, which are resistant to various phosphatases [64]. It can be dephosphorylated after denaturation with guanidine hydrochloride, urea, or formic acid [64]. Tau in PHF is much more extensively phosphorylated than fetal tau both quantitatively and qualitatively, but more importantly, it is assembled into amyloid-like filamentous structures. When I gave a talk at a meeting in Kyoto in 1992, Beyreuther asked me whether the phosphorylation might occur after tau assembly into filaments. That was a very good question.

## 5. Conversion of Normal Tau into Amyloid-Like Fibrils

So, why and how is normal tau converted into PHF? The nature of the initial trigger is unclear, but it is possible that an antiparallel dimer of misfolded tau with β-sheet structure [65] might be generated by dysfunction of protein quality control systems or cellular aging. Then, the misfolded tau may act as a template or seed to convert normal tau into amyloid-like fibrils in a prion-like manner.

**Figure 3:**
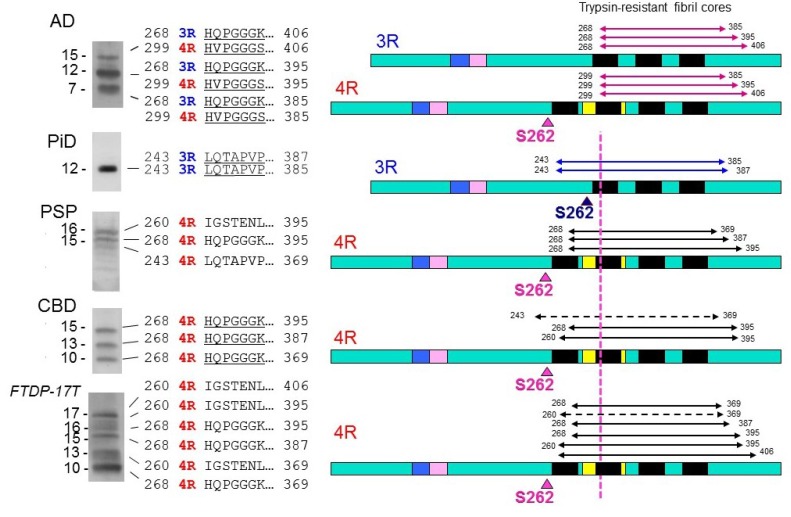
Trypsin-resistant tau bands in tauopathies, the N-terminal sequences of the tau species, and schematic diagrams of the regions in 3R and 4R tau molecules. The major trypsin-resistant regions of tau identified by protein sequencing, MALDI-TOF and LC/MS/MS analyses are shown by solid lines, and minor ones by broken lines. In PiD, Ser262 is in the trypsin-resistant region, which may be buried in the fibril cores, and this may be the reason why Ser262 is not phosphorylated in PiD, whereas it is partially phosphorylated in the other tauopathies.

We biochemically and ultrastructurally investigated abnormal tau proteins in brains with AD and other tauopathies, such as PiD, PSP, CBD, and FTDP-17T (*MAPT* mutation cases with +13 or +16 intron 10 mutation), and confirmed that the major species of abnormal tau deposited in these brains are all six tau isoforms in AD, only 3R tau isoforms in PiD, and only 4R tau isoforms in PSP, CBD and FTDP-17T (with +13 or +16 intron 10 mutation) [54,61]. In addition, we found distinct C-terminal banding patterns of tau in these diseases, except for CBD and FTDP-17T [61,66]. Furthermore, the trypsin-resistant tau banding patterns were highly characteristic, and could be used for biochemical classification of these tauopathies [61]. Since trypsin-resistant tau bands are known to reflect the conformations or structures of the cores of tau fibrils [67], we extensively analyzed them by protein sequencing, LC/MS/MS, and TOF/MS analyses, and found that microtubule binding repeat regions are present in these bands (Figure 3) [61].

Interestingly, the regions involved were slightly different among the diseases. In particular, the amino-terminals of trypsin-resistant tau in AD, in which both 3R tau and 4R tau isoforms are deposited, are clearly distinct from those in 3R tauopathy PiD or 4R tauopathies CBD, PSP and FTDP-17T (intron +13/16) (Figure 3). These results strongly suggest that 3R and 4R tau isoforms are integrated in PHF at a ratio of 1:1 through the microtubule binding repeat regions of tau in AD, whereas only 3R tau isoforms in PiD and only 4R tau isoforms in CBD and PSP are integrated into fibrils through different microtubule binding regions [43,61,68]. The molecular mechanisms remain to be fully clarified but it is suggested that a heterodimer of 3R and 4R tau with antiparallel β-sheet structure is initially generated and converts both 3R and 4R tau into amyloid-like PHFs in AD brain, while a homodimer of 3R or 4R tau with β-sheet structure converts only 3R or 4R tau into amyloid-like fibrils in 3R or 4R tauopathies, respectively [61]. Another important implication of the analysis of trypsin-resistant tau concerns the significance of tau phosphorylation. It has been reported that, exceptionally, S262 is unphosphorylated in PiD, whereas it is partially phosphorylated in the other tauopathies [69,70]. From the analysis of trypsin-resistant tau, Ser262 is in the trypsin-resistant region of tau in PiD, but not in the other tauopathies (Figure 3) [61]. Namely, Ser262 may be buried in the core of tau filaments in PiD, but not in the other diseases. That may be the reason why Ser262 is unphosphorylated in PiD, *i.e.*, kinases cannot access to Ser262 in the core of filaments in PiD. This supports the idea that phosphorylation may be a secondary event after the assembly of tau [61].

**Figure 4:**
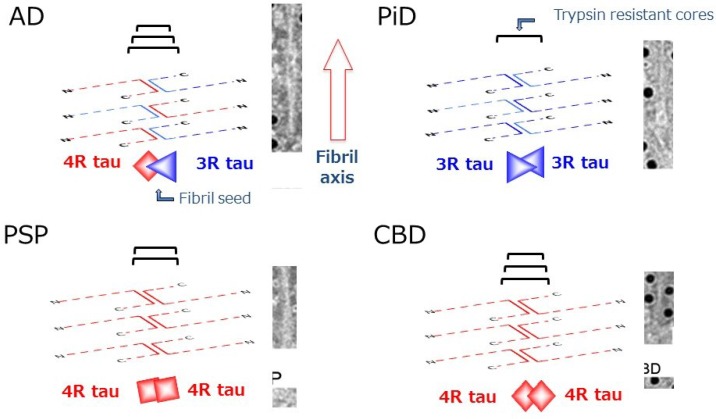
Schematic diagrams of amyloid-like tau fibril formation in tauopathies. In AD, a heterodimer composed of 3R and 4R tau isoforms can seed fibril formation, while only homodimer composed of 3R tau or 4R tau isoforms alone can seed fibril formation in PiD and CBD (or PSP), respectively. The trypsin-resistant tau may represent the core regions of the tau fibrils, which are characteristic for each disease.

The concept of prion-like propagation of abnormal proteins can account for the diverse but well-ordered intracellular amyloid-like fibril formation and spreading in the central and peripheral nervous systems. The pathologies and the spreading may be determined by the kind of tau isoform that is initially misfolded, how it is assembled, and where or in which cells this occurs. These differences may underlie the differences of disease phenotype and progression (Figure 4).

## 6. Molecular Mechanisms of Formation and Propagation of Pathological Tau Proteins

Prion-like seed-dependent tau aggregation has been demonstrated in *in vitro* cellular and mouse models by introduction of preformed tau fibrils [71,72,73,74,75,76]. In the cellular experiments, it has been clearly shown that 3R tau forms aggregate only with abnormal 3R tau fibrils, while 4R tau forms aggregate only with 4R tau fibrils, strongly suggesting that 3R and 4R tau behave as different proteins in terms of their prion-like mechanism [73]. Spreading of the pathology in brains of transgenic mice overexpressing human tau has been demonstrated following injection of brain homogenates containing abnormal tau proteins [74,75]. So, how can these abnormal tau proteins be transmitted from cell to cell? Although several mechanisms, such as classical pathways of endocytosis and exocytosis, travel through exosomes, and transport via tunneling nanotubes, have been discussed [24,77,78,79], it is still not clear how such big molecules can be transmitted from cell to cell, and whether or not the process is receptor-mediated. It should be noted that transmission of pathological tau from mouse to mouse has never been observed so far.

**Figure 5:**
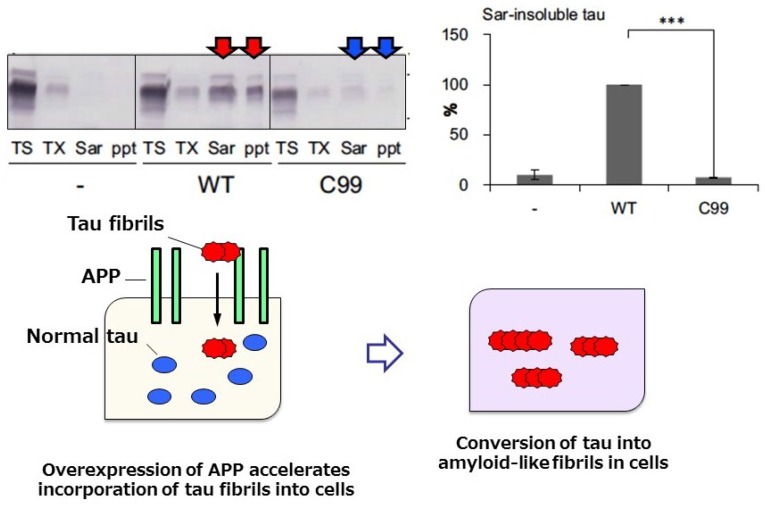
Immunoblot analysis and quantitative analysis of phosphorylated tau in cells overexpressing wild-type APP. Tau accumulation was not observed in cells overexpressing C99 APP lacking the extracellular domain (upper panel), suggesting that the APP extracellular domain is required for interaction with, and incorporation of, tau fibrils. A schematic illustration of tau accumulation in cells expressing APP upon addition of tau fibrils is shown in the lower panel. We investigated the effect of mutations (KM670/671NL, V717F, V717G, or V717I) on tau aggregation, however, significant difference was not detected on tau aggregation in this study. The pathological relevance of these mutations is unclear.

Recently, we found that amyloid protein precursor (APP), mutation of which is involved in familial forms of AD, may work as a receptor of abnormal tau fibrils and promote intracellular tau aggregation [80]. Overexpression of human 3R or 4R tau in cultured cells does not lead to formation of abnormal tau aggregates in the cells, even if preformed tau fibrils are added to the culture, unless transfection reagent is also added. However, cultured cells overexpressing APP formed tau aggregates when tau fibrils were added without any transfection reagent (Figure 5) [80], suggesting that APP on cell membranes may interact with extracellularly added tau fibrils and recruit them into cells.

The interaction was demonstrated by confirming the colocalization of tau fibrils with APP on cell membranes, while no colocalization was observed with normal tau [80]. Next, intracellular accumulation of phosphorylated tau was examined in these cells expressing APP. To distinguish intracellular tau from the tau exogenously added as tau fibrils, HA-tagged tau was expressed and the sarkosyl-insoluble tau extracted from the cells was investigated by immunoblotting and immunoelectron microscopy. We found that AT8 positive-tau fibrils were also positive for anti-HA antibody, indicating that plasmid-derived intracellular tau is accumulated in a phosphorylated and fibrous form; this was also confirmed by immunoblotting [80]. The seeded accumulation of tau was not observed in cells transfecting with a mutant APP (C99) lacking the extracellular domain of APP, suggesting that the extracellular domain is required for binding of APP with tau fibrils (Figure 5) [80]. Thus, APP may be involved in the accumulation and propagation of pathological tau as a receptor of tau fibrils. Previous work on APP has focused on its role as a precursor for Aβ accumulation, but our findings indicate that it may rather play a role in the promotion of tau accumulation. It has also been questioned whether or not there is any link between Aβ pathology to tau pathology; however, we did not detect any effect of Aβ on seeded tau accumulation [80]. Therefore, APP rather than Aβ may accelerate tau accumulation and propagation.

## 7. Conclusions

It has long been debated whether or not Aβ pathology precedes tau pathology in AD, and this has again become a hot topic in AD research [81,82,83,84], as discussed about the primary age-related tauopathy (PART) in a recent issue of Acta Neuropathologica [85,86]. Braak et al. reported that tau pathology develops 20 years before the appearance of Aβ pathology and they found no AD cases showing only Aβ pathology without tau pathology [81]. Therefore, they hypothesized that Aβ may be secreted from the axon terminals of cells with tau pathology [87], namely tau pathology may be the cause of Aβ pathology. This is a very important idea, not only for understanding the pathogenesis of AD, but also for developing therapeutic strategies, because drugs targeted to Aβ are unlikely to be effective on tau aggregation and related neurodegeneration if Aβ are simply secreted from cells with tau pathology. As a result, there is increasing interest in tau-targeted therapies aimed at inhibiting tau fibril formation with chemical compounds [88,89,90,91] and promoting abnormal tau clearance by using antibodies or vaccination [92,93,94]; indeed, tau aggregation inhibitor therapy is already under clinical trial [95]. Since both tau and Aβ pathologies may already be developed by the time patients see a doctor, therapy should be targeted to stop or slow the disease progression. I believe a better understanding of cell-to-cell transmission of tau and its regulation will be the key in the development of disease-modifying drugs for AD.
